# Pre-diagnostic NSAID use but not hormone therapy is associated with improved colorectal cancer survival in women

**DOI:** 10.1038/sj.bjc.6606041

**Published:** 2011-02-08

**Authors:** A E Coghill, P A Newcomb, V M Chia, Y Zheng, K J Wernli, M N Passarelli, J D Potter

**Affiliations:** 1 Fred Hutchinson Cancer Research Center, Cancer Prevention Program, 1100 Fairview Ave N, M4-B402, Seattle, WA 98109, USA

**Keywords:** colorectal cancer survival, COX-2, epidemiology, proximal tumours, NSAIDs, hormone therapy

## Abstract

**Background:**

Non-steroidal anti-inflammatory drugs (NSAIDs) and hormone therapy (HT) independently decrease the risk of colorectal cancer. However, their role in altering survival after a colorectal cancer diagnosis is not well established.

**Methods:**

We examined the association between the use of these common medications before diagnosis and colorectal cancer survival among women in western Washington State diagnosed with incident colorectal cancer from 1997 to 2002. Cases were ascertained using the Surveillance, Epidemiology and End Results cancer registry; mortality follow-up was completed through linkages to the National Death Index. Cox proportional hazards regression was used to estimate hazard ratios (HRs) and 95% confidence intervals (CIs).

**Results:**

We observed no overall association between colorectal cancer survival and pre-diagnostic NSAID use. However, when stratified by tumour sub-site, NSAID use was associated with a reduced risk of colorectal cancer mortality for women diagnosed with proximal (HR=0.55; 95% CI: 0.32–0.92), but not distal or rectal (HR=1.32; 95% CI: 0.83–2.10) tumours. The usage of HT was not associated with colorectal cancer survival overall or by tumour sub-site.

**Conclusion:**

Usage of NSAIDs before diagnosis may be associated with improved colorectal cancer survival among women diagnosed with proximal tumours. The usage of HT does not appear to have a function in altering colorectal cancer mortality.

Non-steroidal anti-inflammatory drug (NSAID) use and hormone therapy (HT) use have each been consistently shown to be associated with a significantly reduced risk of developing colorectal cancer (Giovannucci *et al*, 1994; Newcomb and Storer, 1995; Rossouw *et al*, 2002; Gambacciani *et al*, 2003; Chlebowski *et al*, 2004; Chan *et al*, 2005; Bardia *et al*, 2007; Newcomb *et al*, 2007b; Baron, 2009; Cole *et al*, 2009). However, few studies have investigated the role of these common medications in subsequent mortality after a diagnosis of colorectal cancer.

Only three observational studies in the literature to date have addressed the relationship between NSAID use and colorectal cancer survival (Fuchs and Heseltine *et al*, 2005; Chan *et al*, 2009; Zell *et al*, 2009). A study of stage III colorectal cancer patients (*n*=846 men and women combined) enrolled in a randomized chemotherapy trial observed that consistent aspirin use was associated with 52% lower risk of either cancer recurrence or mortality (Fuchs and Heseltine *et al*, 2005). A report from the Nurses’ Health Study (NHS) and Health Professionals Follow-up Study, (*n*=840 women; 439 men) found that regular aspirin use after a diagnosis of colorectal cancer was associated with a 29% reduced risk of colorectal cancer-specific mortality (Chan *et al*, 2009). Finally, a recent investigation in the California Teachers Study (CTS) cohort (*n*=621 women) observed that regular NSAID use before diagnosis among women was associated with a 42% reduced rate of colorectal cancer mortality (Zell *et al*, 2009).

Three previous studies have observed inverse associations between regular HT use and the risk of death from colorectal cancer (Calle *et al*, 1995; Slattery *et al*, 1999; Mandelson *et al*, 2003), although the design of one study precluded it from distinguishing between the effects of HT on reduced cancer incidence from those of HT on survival after diagnosis (Calle *et al*, 1995). In contrast, results from the Women's Health Initiative and a recent large, population-based study of women with large bowel cancer both observed no association between HT use and colorectal cancer survival (Ritenbaugh *et al*, 2008; Newcomb *et al*, 2009).

We investigated the association between both NSAID use and HT use before cancer diagnosis and subsequent death among female colorectal cases identified from the population-based Surveillance, Epidemiology and End Results (SEER) registry in 13 counties of western Washington State.

## Materials and methods

### Study population

Details of case ascertainment have been published elsewhere (Newcomb *et al*, 2007a, 2007b). Briefly, eligible case subjects included women, aged between 50 and 74 years, residing in 13 counties in western Washington State, who were diagnosed between 1997 and 2002 with incident, invasive colorectal cancer. Women aged between 20 and 49 years diagnosed during the same time period in the Puget Sound counties (3 of the 13) were also eligible for inclusion. Cases were reported to the Cancer Surveillance System, a population-based registry that is part of the National Cancer Institute's SEER program. Eligibility was limited to English-speaking subjects with available telephone numbers. With physician approval, the study subjects received an introductory letter in the mail and were followed-up with a telephone call. A total of 1614 eligible women were identified. Of these cases, 100 were deceased, 151 were lost to follow-up before interview and 181 refused to participate or did not complete the baseline interview, resulting in a final sample size of 1173 cases. Cases were interviewed and enrolled an average of 8.1 months (s.d.=3.2) after colorectal cancer diagnosis. Analyses were restricted to Caucasian women (*n*=1051) because of sparse data among women of other races. The study was approved by the Institutional Review Board of the Fred Hutchinson Cancer Research Center in accordance with assurances filed with and approved by the US Department of Health and Human Services.

### Exposure and covariate assessment

A structured 60-min telephone interview was used to obtain information from all cases on established and potential risk factors for colorectal cancer. Interviewer-collected information included data on history of NSAID and exogenous hormone use, menstrual and reproductive history, smoking history, height and weight, history of colorectal cancer screening, first-degree family history of cancer and demographic factors, such as age and race. For all women, only potential exposures that occurred before a reference date, approximately 2 years before diagnosis, were considered in the analysis. The interview collected information on type and duration of NSAID use (aspirin or ibuprofen) and HT use (oestrogen only or combined oestrogen and progestin).

For NSAIDs, regular use was defined as use at least twice per week for 1 month or greater. Ever use was defined as regular use of any NSAID type at any point in time before the reference date. Never users reported no use or less than the defined regular use threshold before the reference date. Duration of NSAID use was calculated using the reported years of regular use of any type of NSAID medication; information on the reported frequency of use, measured in pills per day on average, was also collected. An NSAID-dose variable used in Cox models was created using both the available duration and frequency information and included the following categories: ⩽1 time per day for ⩽2 years (dose 1); >1 time per day for ⩽2 years (dose 2); ⩽1 time per day for >2 years (dose 3); and >1 time per day for >2 years (dose 4).

For HT, ever use was defined as use of any preparation type for at least 6 consecutive months at any time before the reference date. Never users reported no use or less than the defined ever use threshold before the reference date. Duration of HT use was calculated using the reported years of use of any type of HT preparation.

### Outcome

Vital status on all enrolled cancer cases was determined through linkages to the National Death Index to obtain date and cause of death; cause of death was classified using ICD10 coding conventions. The National Death Index identifies known deaths with a high degree of sensitivity, validity and completeness (Fillenbaum *et al*, 2009). The primary outcome of interest was death due to colorectal cancer. Time to death was evaluated from SEER-recorded date of colorectal cancer diagnosis and National Death Index-recorded date of death. Patients alive at the time of their last known vital assessment were censored at that date, with the most recent vital status linkage occurring in December 2009. Patients dying of causes other than colorectal cancer were censored at their recorded date of death.

Sub-site and stage of the colorectal tumour at diagnosis were defined using SEER records. Advanced disease was defined as colorectal cancer with distant metastasis at diagnosis; non-advanced disease included local and regional stage disease at diagnosis. Sub-site of disease was categorized using ICD10 codes: proximal disease (C18.0–C18.5); distal disease (C18.6–C18.7); and rectal disease (C19.9–C20.9).

### Statistical analysis

Kaplan–Meier survival curves were generated for both NSAID use (ever *vs* never) and HT use (ever *vs* never). The proportional hazards assumption was evaluated graphically as well as statistically through inclusion of interaction terms between exposures and current time in Cox regression models (Ahmed *et al*, 2007). For both exposures investigated, the proportional hazards assumption was not statistically violated for colorectal cancer-specific mortality.

Cox proportional hazards regression models were used to estimate hazard ratios (HRs) and 95% confidence intervals (CIs) for the association between pre-diagnostic NSAID use, HT use and colorectal cancer-specific mortality. To increase comparability to previous studies that excluded metastatic disease (Chan *et al*, 2009), analyses were restricted to cases diagnosed with local or regional disease (*n*=933). Cox regression models included the following list of covariates selected *a priori*: age at cancer diagnosis, body mass index at reference date, smoking status, family history of colorectal cancer, history of preventive screening and stage of disease at diagnosis. Additionally, the regression models investigating each medication were adjusted for pre-diagnostic use of the other medication (i.e., NSAID use adjusted for pre-diagnostic HT use). Preventive screening was defined as sigmoidoscopy or colonoscopy (endoscopy) screening that was received at least 2 years before the diagnosis of colorectal cancer. Categories of body mass index were defined as the following in units of kg m^−2^: not overweight <25.0, overweight 25.0–29.9, obese ⩾30.0 (Clinical Guidelines on the Identification, Evaluation, and Treatment of Overweight and Obesity in Adults, 1998).

The multivariable Cox regression models were evaluated across strata of tumour sub-site (proximal *vs* distal/rectal). Statistical interaction between NSAID use, HT use and tumour sub-site was investigated by the inclusion of interaction terms between the respective medication use (yes/no) and tumour sub-site (proximal/distal, rectal) in regression models. All statistical analyses were performed using SAS software, version 9.1 (SAS Institute Inc., Cary, NC, USA); all *P*-values reported are two sided.

## Results

After an average of 6.3 years of follow-up after cancer diagnosis and study enrolment, a total of 371 deaths from all causes and 274 deaths from colorectal cancer were ascertained. Among women with non-advanced colorectal disease, 266 deaths from all causes and 149 deaths specifically due to colorectal cancer were identified.

Approximately 50% of the women in the study population reported ever using NSAIDs before their cancer diagnosis. Among women at least 50 years of age, the proportion of ever HT users was slightly higher than 55% (Table 1). Ever users of NSAIDs, compared with never users, were more likely to have a family history of colorectal cancer, have a history of preventive screening, and to be obese. Although ever users of HT were also more likely than never users to have a history of preventive screening, they were less likely to be obese and showed no substantive differences in previous family history of colorectal cancer compared with never users. Greater proportions of both ever users of NSAIDs and ever users of HT were diagnosed with localised tumours.

**Figure 1:**
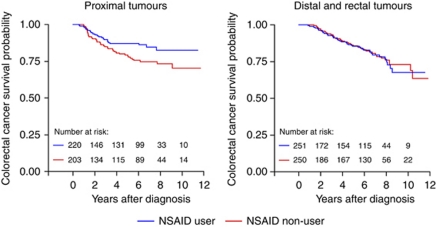
Colorectal cancer survival according to pre-diagnostic NSAID use among women.

The HR for colorectal cancer survival after diagnosis associated with pre-diagnostic NSAID use was 0.88 (95% CI: 0.62–1.24) (Table 2). We observed evidence of heterogeneity in the association of NSAID use before diagnosis with colorectal cancer survival according to the sub-site of the diagnosed colorectal tumour (*P*-value for interaction=0.03). Pre-diagnostic NSAID use was significantly associated with improved colorectal cancer survival among cases diagnosed with proximal disease (HR=0.55; 95% CI: 0.32–0.92). In contrast, we observed no apparent association with survival among women diagnosed with left-sided disease (Table 2). This null association was observed among subgroups of left-sided disease, including cases diagnosed with distal tumours (HR=1.40; 95% CI: 0.62–3.19) and cases diagnosed with rectal tumours (HR=1.25; 95% CI: 0.70–2.22). Colorectal cancer survival curves, based on NSAID use (ever *vs* never), for both proximal tumours and distal/rectal tumours, are presented in Figure 1.

The statistically significant reduction in the risk of colorectal cancer mortality among proximal cases associated with pre-diagnostic NSAID use was observed to be dose dependent (*P*-trend=0.01). Although women who reported use on average not more than once per day for 2 years or less (dose 1) were not observed to experience a reduced risk of colorectal cancer mortality (HR: 1.06; 95% CI: 0.50–2.26), women who used NSAIDs more than once a day for greater than 2 years (dose 4) experienced approximately one quarter the risk of dying of colorectal cancer (HR: 0.26; 95% CI: 0.07–0.88) compared with never users (data not shown).

Estimates for aspirin and ibuprofen use were not observed to be of similar magnitude, with a much stronger association present among regular ibuprofen users. However, the limited number of cases regularly using ibuprofen precludes any definitive conclusions, and these type-specific differences should be investigated further in studies adequately power to examine associations within subgroups of NSAID type. The patterns of regular use for aspirin and ibuprofen also differed substantially. Ibuprofen users appeared to be using at higher doses (pills per day) on a more regular basis than aspirin users (data not shown); the pattern of use and not type of NSAID may, therefore, potentially account for observed differences.

Pre-diagnostic HT use was not associated with the risk of colorectal cancer mortality among cases (HR: 0.95; 95% CI: 0.66–1.35). The association between pre-diagnostic HT use and colorectal cancer survival was consistently null among cases reporting use of oestrogen-only preparations as well as cases reporting use of oestrogen plus progestin preparations. Additionally, the association between pre-diagnostic HT use and colorectal cancer survival was null among cases diagnosed with both proximal tumours and those diagnosed with distal or rectal tumours. Longer durations of HT use were also not observed to alter the risk of colorectal cancer mortality after diagnosis. Finally, we did not observe any significant heterogeneity in the association of HT with colorectal cancer survival according to NSAID use status (*P*-value for interaction=0.29).

## Discussion

We observed an inverse association between NSAID use and death due to colorectal cancer among Caucasian women diagnosed with proximal disease. Women who regularly used NSAIDs before diagnosis experienced approximately half the risk of colorectal cancer mortality compared to never users. These women experienced an approximate halving of risk of colorectal cancer mortality compared with never users. This association was observed to be dose-dependent, with reductions in colorectal cancer mortality of greater magnitude associated with increasing amounts of NSAID use. We did not, however, observe any evidence of an association between HT use before diagnosis and colorectal cancer survival, regardless of hormone preparation type, duration of use or tumour characteristics at diagnosis.

Previous studies provide evidence that NSAID use may be important for colorectal cancer survival after diagnosis (Fuchs and Heseltine *et al*, 2005; Chan *et al*, 2009; Zell *et al*, 2009). The report from the California Teachers Study observed a dose-dependent improvement in colorectal cancer survival associated with NSAID use before diagnosis (Zell *et al*, 2009). The NHS and Health Professionals Follow-up Study (Chan *et al*, 2009) reported that any association with improved survival was limited to post-diagnostic NSAID use. However, cases from the NHS and Health Professionals Follow-up Study cohorts did not experience any significant survival benefit associated specifically with post-diagnostic use if they also reported aspirin use before their colorectal cancer diagnosis (HR: 0.89; 95% CI: 0.59–1.35). These findings suggest that NSAID use both before and after diagnosis may have an important role in altering colorectal cancer survival.

Previous studies have not reported potential differences in the association between NSAID use and colorectal cancer mortality according to tumour sub-site. The NHS and Health Professionals Follow-up Study examined potential differences according to whether the tumour was located in the colon *vs* the rectum and observed no heterogeneity. Our study is the first to report results according to proximal *vs* distal or rectal tumour location, making comparison of our results to previous survival studies difficult. However, previous literature has reported a site-specific effect for NSAIDs on the risk of developing incident colorectal cancer (Rosenberg *et al*, 1991; Smalley *et al*, 1999; Mahipal *et al*, 2006), with a stronger inverse association being observed for proximal disease.

Our findings for HT are consistent with results from the Women's Health Initiative, as well as data from a large population-based study of large bowel cancer, both of which showed no overall association between HT use and colorectal cancer survival (Ritenbaugh *et al*, 2008; Newcomb *et al*, 2009). Previous studies that observed a statistically significant reduction in colorectal cancer mortality associated with HT often failed to account for important covariates, such as colorectal cancer screening or NSAID use in their analyses (Slattery *et al*, 1999; Mandelson *et al*, 2003). We did in fact observe different prevalences of both screening and NSAID use among HT users compared with never users in our study, highlighting the importance of accounting for these potential confounders in analyses to obtain an unbiased estimate of the effect of HT use before diagnosis on colorectal cancer survival.

There are several plausible mechanisms by which NSAID use before diagnosis could affect colorectal cancer mortality among women. Prostaglandin synthases COX-1 and COX-2 are directly inhibited by NSAIDs, altering prostaglandin production and cellular inflammatory responses (Sheng *et al*, 1997; Brown and DuBois, 2005; Rodriguez-Moranta and Castells, 2005; Wang and Dubois, 2010). This alteration of prostaglandin production results in the promotion of apoptosis, inhibition of angiogenesis and disruption of processes crucial to tumour growth (Sheng *et al*, 2001; Chan, 2006; Wang and Dubois, 2006; Cha and DuBois, 2007; Greenhough *et al*, 2009). The anti-inflammatory effects of NSAID medications may also impact upon colorectal cancer survival directly as inflammation has been linked to both colorectal cancer incidence and cancer progression (Coussens and Werb, 2002; Erlinger *et al*, 2004; Kim *et al*, 2008; Mantovani *et al*, 2008), and multiple markers of systemic inflammation have been linked specifically to colorectal cancer prognosis (Ishizuka *et al*, 2007; Shiu *et al*, 2008; Roxburgh *et al*, 2009).

The use of NSAIDs before diagnosis may not only decrease the likelihood of an inflammatory tumour micro-environment but also may alter the type of tumour that initially develops. The inhibition of the COX-2 pathway may be of particular importance; multiple studies have observed that COX-2 expression is a negative prognostic factor for patients with colorectal cancer and results in high colorectal tumour loads in animal studies (Masunaga *et al*, 2000; Soumaoro *et al*, 2004; Yamac *et al*, 2005; Ogino *et al*, 2008). An earlier report from the NHS observed that regular pre-diagnostic aspirin use resulted in a lower than expected incidence of tumours expressing high levels of COX-2 (Chan *et al*, 2007). Use of NSAIDs before diagnosis may lead to the development of tumours expressing lower levels of COX-*2*, resulting in the diagnosis of tumours that are less aggressive and have a molecular profile that improves survival.

Proximal colorectal tumours have a distinct molecular phenotype compared with distal or rectal tumours (Richman and Adlard, 2002; Azzoni *et al*, 2007; Benedix *et al*, 2010) and may, therefore, represent a different form of colorectal disease. For example, variation in expression of the NSAID-targeted COX-2 enzyme according to the location of colorectal tissue has been described previously (Dimberg *et al*, 1999; Wiese *et al*, 2003; Nasir *et al*, 2004), and may be a potential explanation for our observed tumour sub-site difference. Additionally, tumours that feature a high-degree of CpG island methylation are more likely to be found in the proximal colon, particularly among women (Jass, 2007). Chronic inflammation has previously been hypothesised to accelerate the process of methylation in patients with ulcerative colitis (Issa *et al*, 2001), providing a possible aetiologic link between inflammatory processes and proximal colorectal tumours. Although the exact molecular mechanisms behind this ‘proximal effect’ are not presently clear, its implications have growing importance in light of recent reports that have shown that proximal tumours have inherently poorer prognosis than more distal tumours (Wray *et al*, 2009; Benedix *et al*, 2010).

This study's strengths included having a large, population-based sample of women and the collection of detailed exposure information for both NSAIDs and HT, including data on type, duration and frequency of NSAID medication used, as well as information on HT preparation type and duration of use. Follow-up of all enrolled colorectal cancer cases was complete and standardized. The inclusion of important covariates in our regression models allowed us to generate measures of association that were not biased by potentially strong confounders such as screening. Additionally, the availability of data on tumour characteristics at diagnosis allowed us to examine differences in hypothesised associations across tumour sub-site.

Limitations of the study include the availability of only pre-diagnostic information for NSAIDs and HT. It seems likely that pre-diagnostic medication use is correlated with post-diagnostic use, and future studies to examine these exposures independently are warranted. Information on medication use was collected after diagnosis, introducing the potential for incorrect recall, although this likely affected all cases equally and as such would result in non-differential misclassification and bias of the observed association to the null. Only limited treatment information was available in this population; however, SEER-reported first-course treatment type mirrored stage of disease at diagnosis for the majority of cases, and we accounted for extent of disease in our analyses. We did observe that after accounting for stage of disease at diagnosis, women diagnosed with rectal tumours were slightly more likely to receive treatment (data not shown). Finally, we were unable to enroll all cases immediately after diagnosis. Failure to enroll cases in a timely manner can result in patient loss that may lead to a lack of representativeness of the study cohort (Coghill *et al*, 2009). However, we did not experience a high percentage of patient loss, as we were able to enroll approximately 80% of cases within 10 months of their diagnosis.

In addition to colorectal cancer-specific survival, we attempted to explore the association between the use of these common medications before diagnosis and all-cause mortality. We observed HRs similar in direction to those reported for colorectal cancer mortality, with no significant associations observed. However, the vast majority of deaths in our cohort were due to colorectal cancer, limiting our ability to investigate the relationship with mortality from other causes.

Despite the inverse association consistently observed between NSAIDs and HT in relation to colorectal cancer incidence in women, our results suggest that this same relationship did not uniformly extend to colorectal cancer mortality. Pre-diagnostic NSAID use was associated with significantly improved colorectal cancer survival among women diagnosed with proximal tumours. However, HT was not associated with colorectal cancer survival. Future studies should examine molecular characteristics of diagnosed tumours, including COX-2 expression and methylation status among regular NSAID users, and the potential interaction of NSAID use history with patient characteristics, such as genetic polymorphisms. Studies of the use of these common medications in relation to survival after a diagnosis of colorectal cancer should also be conducted among multi-ethnic cohorts of women. The practical implication of this study is that regular NSAID use earlier in life in persons at risk of colorectal cancer may not only prevent disease but also may prevent more aggressive disease. This should be considered when weighing the risks and benefits of NSAID use as a chemopreventive therapy.

## Figures and Tables

**Table 1:** Characteristics of study women (*n*=1051) according to pre-diagnostic NSAID and HT use

	**NSAID use** a				**Hormone therapy** b			
	**Never (*n*=507)**		**Ever (*n*=534)**		**Never (*n*=401)**		**Ever (*n*=519)**	
	***n***	**%**	***n***	**%**	***n***	**%**	***n***	**%**
*Age at diagnosis*								
<50 years	59	11.6	61	11.4	—	—	—	—
50–59 years	145	28.6	123	23.0	107	26.7	160	30.8
60–69 years	176	34.7	210	39.3	148	36.9	237	45.7
>70 years	127	25.0	140	26.2	146	36.4	122	23.5
								
*BMI (kg *m^−2^*)*								
<25.0	231	45.6	193	36.1	146	36.4	209	40.3
25.0–29.9	156	30.8	157	29.4	119	29.7	168	32.4
⩾30.0	118	23.4	183	34.3	135	33.8	141	27.2
								
*Menopausal status* b								
Pre-menopausal	25	5.6	17	3.6	30	7.5	13	2.5
Natural menopause	245	54.7	223	47.2	252	62.8	216	41.7
Induced/other menopause	178	39.7	232	49.2	119	29.7	289	55.8
								
*Family history of colorectal cancer*								
No	430	84.8	431	80.7	331	82.5	432	83.2
Yes	77	15.2	103	19.3	70	17.5	87	16.8
								
*Smoking status*								
No	227	44.8	247	46.3	180	44.9	244	47.0
Former	183	36.1	211	39.5	149	37.2	197	38.0
Current	97	19.1	76	14.2	72	18.0	78	15.0
								
*Preventive screening*								
No	470	90.7	475	88.0	373	93.0	461	88.8
Yes	37	7.3	59	11.0	28	7.0	58	11.2
								
*Tumour sub-site*								
Proximal	224	44.2	255	47.8	185	46.1	257	44.4
Distal	133	26.2	132	24.7	111	27.7	121	23.3
Rectal	150	29.6	147	27.5	105	26.2	141	27.2
								
*Stage of disease at diagnosis*								
Localized	193	38.3	234	43.8	162	40.5	230	44.5
Regional	257	51.0	237	44.4	184	46.0	242	46.8
Distant	54	10.7	63	11.8	54	13.5	45	8.7

Abbreviations: BMI=body mass index, HT=hormone therapy, NSAID=non-steroidal anti-inflammatory drug.

a Not all women responded to the interview questions regarding medication use, resulting in missing data on NSAID use and HT use for *n*=10 women.

b Percentages and counts restricted to women at least 50 years of age (*n*=930).

**Table 2:** HRs and 95% CIs for the association between NSAID and HT use before diagnosis and colorectal cancer mortality, stratified by tumour sub-site

	**All tumour sub-sites combined**				**Proximal tumours**		**Distal/rectal tumours**	
**Medication exposure**	**Cases at risk** a	**Colorectal cancer deaths**	**HR**	**95% CI**	**HR**	**95% CI**	**HR**	**95% CI**
*NSAID use* b								
Never	444	81	1.00	Referent	1.00	Referent	1.00	Referent
Ever	466	62	0.88	0.62–1.24	0.55	0.32–0.92	1.32	0.83–2.10
Aspirin^c^	340	50	1.14	0.77–1.69	0.75	0.41–1.37	1.64	0.96–2.78
Ibuprofen	182	19	0.51	0.26–1.00	0.09	0.01–0.69	0.94	0.45–1.97
								
*HT use* d								
Never	344	57	1.00	Referent	1.00	Referent	1.00	Referent
Ever	465	68	0.95	0.66–1.35	0.90	0.53–1.54	0.95	0.57–1.56
Oestrogen only	429	60	1.04	0.69–1.55	1.17	0.65–2.08	0.91	0.51–1.63
Oestrogens and progestin	206	26	0.92	0.43–1.97	0.72	0.17–3.16	1.19	0.46–3.09

Abbreviations : CI=confidence interval; HR=hazard ratio; HT=hormone therapy; NSAID=non-steroidal anti-inflammatory drug.

Models in table above are adjusted for age at diagnosis, body mass index, smoking status, family history of colorectal cancer, history of preventive screening and stage of disease at diagnosis.

a Reported number of cases at risk and number of colorectal cancer deaths are out of the total number of Caucasian women with non-advanced disease (*n*=933) for each exposure. All HT models are restricted to Caucasian women at least 50 years of age with non-advanced disease (*n*=818). Note that not every case answered the medication questions in the interview.

b Additionally adjusted for HT use (never, ever).

c Results by type are examined as ever use, adjusted for use of other medication types. For example, results reported for aspirin are generated from a model comparing ever users of aspirin to never users, including ‘ever use’ of ibuprofen in the model.

d Additionally adjusted for NSAID use (never, ever).
